# Clinical features and prognostic factors of pulmonary carcinosarcoma: A nomogram development and validation based on surveillance epidemiology and end results database

**DOI:** 10.3389/fmed.2022.988830

**Published:** 2022-10-18

**Authors:** Ming-Yi Zhang, Lian-Sha Tang, Zhao-Juan Qin, Ya-Ting Hao, Ke Cheng, Ai Zheng

**Affiliations:** ^1^Department of Obstetrics and Gynecology, West China Second University Hospital, Sichuan University, Chengdu, Sichuan, China; ^2^Department of Biotherapy, Cancer Center, West China Hospital, Sichuan University, Chengdu, Sichuan, China; ^3^School of Clinical Medicine, Ningxia Medical University, Yinchuan, Ningxia, China

**Keywords:** rare lung malignant disease, pulmonary carcinosarcoma, the surveillance epidemiology and end results, propensity-score matching, nomogram, prognosis

## Abstract

**Background:**

Pulmonary carcinosarcoma (PCS) is a rare but aggressive malignant disease in the lung. It is characterized by coexisting histologic elements of carcinomatous and sarcomatous components. This study aimed to comprehensively understand the clinical features of PCS and develop a nomogram for prognostic prediction of PCS patients.

**Methods:**

Data were collected from the Surveillance Epidemiology and End Results (SEER) database between 1975 and 2018. Propensity-score matching (PSM) was used to match the demographic characteristic of the PCS vs. pulmonary sarcoma (PS). Cancer-specific survival (CSS) and overall survival (OS) were the main endpoints of the survival of patients and were evaluated using the Kaplan Meier curves and Cox proportional hazards regression. We further randomly split enrolled PCS patients from SEER into the training and validation sets. All independent predictors for OS of the training set were integrated to create a predictive nomogram. The performance of the nomogram was determined by discrimination, calibration ability, clinical usefulness, and risk stratification ability both in the training and validation cohorts. In addition, the clinical data of PCS patients from the West China Hospital were also retrospectively analyzed by this model.

**Results:**

A total of 428 PCS patients and 249 PS patients were enrolled from SEER. Compared to pure PS, PCS was associated with significantly better survival in the unmatched cohorts, whereas non-significantly better survival after PSM. In subgroup analysis, PCS showed significantly worse survival than pure PS in subgroups among the race, marital status, and radiation treatment. A nomogram was constructed for PCS patients’ survival prediction by combining the independent risk factors, including gender, stage, surgery, radiation, and chemotherapy. The nomogram showed good discrimination, calibration, and predictive power in the training and validation sets. Risk stratification analysis indicated that the nomogram scores efficiently divided PCS patients into low and high-risk groups.

**Conclusion:**

PCS is a rare malignant disease of the lung with distinct clinical features. It had a comparable survival compared with pure PS in the matched cohorts. In addition, a nomogram was developed and validated for predicting the OS in PCS patients.

## Introduction

Pulmonary carcinosarcoma (PCS) is a rare malignant disease of the lung (less than 1% of all primary pulmonary neoplasms) that was first reported by Kika in 1908 (1–4). According to the newest 2021 World Health Organization (WHO) classification of thoracic tumors, PCS was a subtype of sarcomatoid carcinoma (constituting a group of spindle-cell carcinoma, pleomorphic carcinoma, giant-cell carcinoma, pulmonary blastema, and carcinosarcoma) (5). PCS is defined by a biphasic histopathological pattern consisting of carcinomatous epithelial and sarcomatous mesenchymal elements (6, 7). The squamous cell carcinoma, followed by adenocarcinoma and large cell carcinoma, is reported to be the most common carcinomatous component, whereas poorly differentiated spindle cell sarcoma, chondrosarcoma, and osteosarcoma be the more often sarcomatous component (8–10).

Due to its rarity, PCS patients’ clinical characteristics, treatment, and survival outcomes are poorly defined. Most existing literature has involved only a small case series or a small number of retrospective studies. It seems that PCS tends to present in middle age, with the average age of diagnosis around 60 years, and is more common among males with a heavy smoking history (11–13). Since sarcomas are usually resistant to radiation and chemotherapy, like pulmonary sarcoma (PS), complete surgical resection is also considered the most optimal treatment choice for PCS patients (11, 14, 15). For what concerns the prognosis of these patients, limited available studies have reported conflicting results. One study reported that PCS’s 5-year OS rate is 49.3% and a mean survival time of 37.1 months, which is no significant difference compared to pure PS (48.8% and 37.8 months) (13). However, the other two studies estimated the 5-year OS rate for PCS is only 21.3 or 22.7% (16, 17).

On the predictors of prognosis for PCS, though tumor diameter, invasive peripheral type, and lymph node metastasis were reported that might affect the prognosis negatively, there is still a lack of effective models for accurate prediction (18, 19). Nomogram is a continuously visualized calculation or algorithm to predict a particular clinical event by integrating multiple correlated variables, which was superior to the traditional model and system (20, 21). In this study, the clinical features of PCS patients from the Surveillance Epidemiology and End Results (SEER) database were analyzed and compared with pure PS using propensity-score matching (PSM) to minimize confounding factors. Then a nomogram for predicting the prognosis of PCS patients was developed to guide the selection of clinical treatment options.

## Materials and methods

### Study population

Data were extracted from the SEER*Stat software version 8.4.1.^1^ The SEER database was reviewed to identify patients with PCS and pure PS between the years 1975 and 2018. Inclusion criteria were as follows: (I) site recode ICD-O-3 (International Classification of Diseases for Oncology, 3rd Edition) was restricted to “Lung and Bronchus’; (II) pathologically confirmed carcinosarcoma [ICD-O-3 (8980/3)], carcinosarcoma, embryonal type [ICD-O-3 (8981/3)], or sarcoma [ICD-O-3 (8800/3)]; (III) known age and survival data. Patients with undetermined positive histology or first primary tumors were excluded. The clinical data of patients with pathologically diagnosed primary PCS in the West China Hospital from 2006 to 2022 were also collected.

### Study variables

Clinical variables of enrolled patients included age, gender, race, marital status, tumor staging and grading, surgery, radiation, and chemotherapy. Cancer-specific survival (CSS) and overall survival (OS) were the main endpoints of the survival of patients. CSS was defined as the interval between cancer diagnosis and death from lung cancer. OS was assessed as the period from the cancer diagnosis to death resulting from any cause or the last follow-up.

### Statistical analysis

We used descriptive statistics to summarize the baseline characteristics of these rare lung diseases. Categorical variables were shown as frequencies and proportions. Chi-squared test tested differences in characteristics by categorization. To minimize confounding factors, we used random 1:1 nearest-neighbor PSM without replacement to balance all baseline covariates between PCS and PS. CSS and OS were estimated using the Kaplan-Meier method and compared with the log-rank test. Univariate and multivariable Cox regression analyses were used to evaluate the impact of clinical variables. The HRs and 95% CIs were also calculated.

To construct and validate a nomogram for PCS patients’ OS prediction, we further randomly split enrolled PCS patients from SEER into the training (*n* = 300) and validation sets (*n* = 128) in a 7:3 ratio. For the training set, univariate and multivariate Cox regression analyses were used to determine the independent prognostic factors of OS. Variables with *p* < 0.05 in the multivariable analyses were selected for the final nomogram development of predicting 1, 2, 3, and 5-years OS for PCS patients. The performance of the nomogram in the training and validation sets was assessed as follows: The concordance index (C-index) was used to quantify discrimination. We also drew a calibration curve to determine the consistency between actual prognosis and predicted survival. The area under the receiver operator characteristic curve (ROC) was calculated to show the model’s predictive power. After obtaining the risk probability from the nomogram, the PCS patients from SEER were divided into the low- and high-risk groups according to the median value of the risk scores. The median OS of different risk groups from the training and validation sets were estimated using the Kaplan-Meier method and compared with the log-rank test, respectively. In addition, the clinical records of 25 patients with pathologically diagnosed PCS in the West China Hospital from 2006 to 2022 were also retrospectively analyzed by this model. Data analyses were conducted using the SPSS Statistics version 25 (IBM Corporation, Armonk, NY, USA) and RStudio version 1.4.1106 (RStudio, Boston, MA, USA). A two-sided *p*-value < 0.05 was considered statistically significant.

## Results

### Patient characteristics

**FIGURE 1:**
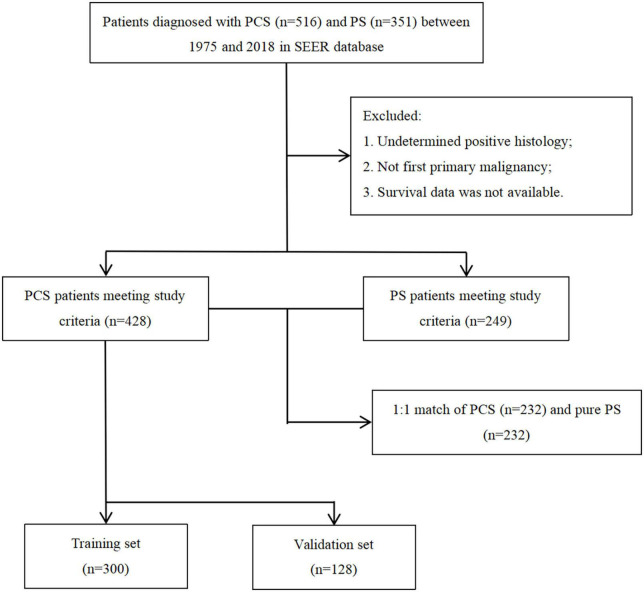
Patient selection scheme for the study.

A total of 516 PCS patients and 351 PS patients were extracted from the SEER database. After removing patients with unavailable necessary information, 428 PCS and 249 pure PS patients were included in this analysis. The patients’ screening flowchart is shown in Figure 1. Approximately 65.8% of PCS patients were aged 60–80 at diagnosis, and the proportion of male PCS patients (58.6%) was more than females. We found a more regional (32.9 vs. 18.8%) but less distant stage (26.1 vs. 40.5%) in patients with PCS histology compared to patients with pure PS. Additionally, the PCS group presented a higher proportion of patients who did receive surgery compared with PS (64 vs. 39.3%). On the other hand, the proportion of patients who underwent radiation or chemotherapy in the PCS group was similar to pure PS.

Throughout PSM analysis, these patients were matched 1:1 by age, gender, race, marital status, tumor staging and grading, and treatment. All covariates were subsequently well balanced in the 1:1 matched cohort of PCS (*n* = 232) and PS (*n* = 232). The clinical features of these patients are summarized in Table 1.

**TABLE 1:** Clinicopathologic characteristics of PCS and PS patients in unmatched and matched cohorts.

Characteristics	Unmatched			1:1 matched by PSM		
		
	PCS (*n* = 428)	PS (*n* = 249)	*p*_1_-value	PCS (*n* = 232)	PS (*n* = 232)	*p*_2_-value
Age (years)			<0.001			<0.001
<60	105 (24.53%)	84 (33.73%)		33(14.22%)	71 (30.60%)	
60–80	282 (65.89%)	127 (51.00%)		183 (78.88%)	126 (54.31%)	
>80	41 (9.58%)	38 (15.26%)		16 (6.90%)	35 (15.09%)	
Race			0.313			0.746
White	359 (83.88%)	201 (80.72%)		194 (83.62%)	189 (81.47%)	
Black	47 (10.98%)	28 (11.24%)		24 (10.34%)	25 (10.78%)	
Other/unknown	22 (5.14%)	20 (8.03%)		14 (6.03%)	18 (7.76%)	
Gender			0.54			0.222
Female	177 (41.36%)	97 (38.96%)		105 (45.26%)	92 (39.66%)	
Male	251 (58.64%)	152 (61.04%)		127 (54.74%)	140 (60.34%)	
Marital status			0.253			0.55
Married	358 (83.64%)	211 (84.74%)		197 (84.91%)	197 (84.91%)	
Unmarried	59 (13.79%)	27 (10.84%)		28 (12.07%)	24 (10.34%)	
Unknown	11 (2.57%)	11 (4.42%)		7 (3.02%)	11 (4.74%)	
Stage			<0.001			0.005
Localized	77 (17.99%)	47 (18.88%)		52(22.41%)	46 (19.83%)	
Regional	141 (32.94%)	47 (18.88%)		73 (31.47%)	46 (19.83%)	
Distant	112 (26.17%)	101 (40.56%)		59 (25.43%)	89 (38.36%)	
Unknown	98 (22.90%)	54 (21.69%)		48 (20.69%)	51 (21.98%)	
Grade recoded			<0.001			0.047
I-II	11 (2.57%)	15 (6.02%)		10(4.31%)	10 (4.31%)	
III-IV	183 (42.76%)	142 (57.03%)		104 (44.83%)	130 (56.03%)	
Unknown	234 (54.67%)	92 (36.95%)		118 (50.86%)	92 (39.66%)	
Surgery			<0.001			<0.001
No/unknown	154 (35.98%)	151 (60.64%)		78(33.62%)	134 (57.76%)	
Yes	274 (64.02%)	98 (39.36%)		154 (66.38%)	98 (42.24%)	
Radiation			0.371			0.848
No/unknown	276 (64.49%)	152 (61.04%)		143 (61.64%)	145 (62.50%)	
Yes	152 (35.51%)	97 (38.96%)		89 (38.36%)	87 (37.50%)	
Chemotherapy			0.355			0.921
No/unknown	272 (63.55%)	167 (67.07%)		155 (66.81%)	156 (67.24%)	
Yes	156 (36.45%)	82 (32.93%)		77 (33.19%)	76 (32.76%)	

Data were expressed number (percentage).

### Survival analysis of pulmonary carcinosarcoma and pure pulmonary sarcoma

**FIGURE 2:**
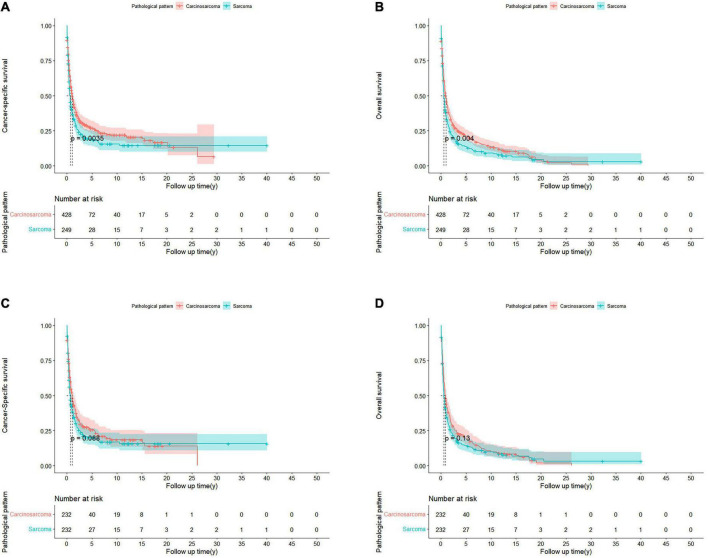
Kaplan-Meier curves of patients with PCS and PS in unmatched and matched cohorts. Before PSM, CSS **(A)** and OS **(B)** in patients with PCS and PS. After PSM, CSS **(C)** and OS **(D)** in PCS 1:1 matched with PS cohorts.

For the cohorts before PSM, PCS patients had significantly better CSS (*p* = 0.0035) and OS (*p* = 0.004) than pure PS. The median CSS of the PCS and PS group were 1.06 and 0.58 years, respectively, whereas the median OS of the PCS and PS group were 0.92 and 0.56 years, respectively (Figures 2A,B).

However, after PSM, the median CSS of PCS patients was 1.05 years, which was no longer a statistically significant difference compared with the PS patients (0.61 years; *p* = 0.088; Figure 2C). The same trend was observed in the OS of the two groups, with the median OS of 0.75 and 0.53 years for PCS and pure PS patients, respectively (*p* = 0.13; Figure 2D). In the Cox proportional hazards regression of matched PCS and pure PS patients (Table 2), pure PS group was also not associated with statistically worse CSS (HR 0.94; 95% CI: 0.75–1.18; *p* = 0.586) and OS (HR 0.96; 95% CI: 0.77–1.18; *p* = 0.688).

**TABLE 2:** Univariate and multivariate Cox analyses of CSS or OS for PSC and PS patients in matched cohorts.

Characteristics	CSS for PCS and PS				OS for PCS and PS			
		
	Univariable		Multivariable		Univariable		Multivariable	
				
	HR (95%)	*P*	HR (95%)	*P*	HR (95%)	*P*	HR (95%)	*P*
**Pathological pattern**								
Carcinosarcoma	1		1		1		1	
Sarcoma	1.19 (0.97, 1.47)	0.0976	0.94 (0.75, 1.18)	0.5864	1.16 (0.95, 1.40)	0.1408	0.96 (0.77, 1.18)	0.6883
**Age (years)**								
<60	1		1		1		1	
60–80	1.27 (0.98, 1.65)	0.0720	1.26 (0.95, 1.66)	0.1103	1.39 (1.09, 1.77)	0.0083	1.33 (1.02, 1.72)	0.0341
>80	1.90 (1.29, 2.80)	0.0011	1.34 (0.88, 2.04)	0.1762	2.12 (1.49, 3.02)	<0.0001	1.54 (1.05, 2.27)	0.0276
**Race**								
White	1		1		1		1	
Black	0.93 (0.65, 1.32)	0.6734	0.87 (0.60, 1.27)	0.4740	0.97 (0.71, 1.33)	0.8501	0.93 (0.67, 1.30)	0.6775
Other/unknown	1.27 (0.85, 1.90)	0.2363	1.37 (0.91, 2.06)	0.1344	1.23 (0.84, 1.79)	0.2908	1.25 (0.85, 1.85)	0.2510
**Gender**								
Female	1		1		1		1	
Male	1.33 (1.07, 1.65)	0.0093	1.20 (0.96, 1.51)	0.1125	1.45 (1.19, 1.77)	0.0003	1.31 (1.06, 1.61)	0.0120
**Marital status**								
Married	1		1		1		1	
Unmarried	0.81 (0.57, 1.14)	0.2237	0.96 (0.67, 1.36)	0.8104	0.77 (0.56, 1.05)	0.1008	0.93 (0.67, 1.29)	0.6651
Unknown	0.50 (0.26, 0.97)	0.0406	0.48 (0.24, 0.96)	0.0375	0.54 (0.31, 0.94)	0.0285	0.51 (0.28, 0.90)	0.0212
**Stage**								
Localized	1		1		1		1	
Regional	2.82 (1.96, 4.05)	<0.0001	2.65 (1.82, 3.87)	<0.0001	2.07 (1.54, 2.79)	<0.0001	1.99 (1.45, 2.72)	<0.0001
Distant	5.84 (4.11, 8.29)	<0.0001	4.68 (3.16, 6.92)	<0.0001	4.02 (3.01, 5.37)	<0.0001	3.19 (2.29, 4.45)	<0.0001
Unknown	2.49 (1.72, 3.61)	<0.0001	2.59 (1.75, 3.82)	<0.0001	1.60 (1.18, 2.19)	0.0027	1.65 (1.19, 2.30)	0.0028
**Grade recoded**								
I–II	1		1		1		1	
III–IV	2.41 (1.27, 4.56)	0.0069	1.52 (0.79, 2.95)	0.2112	1.97 (1.18, 3.29)	0.0096	1.24 (0.73, 2.11)	0.4340
Unknown	1.78 (0.94, 3.38)	0.0768	1.23 (0.64, 2.38)	0.5288	1.50 (0.90, 2.50)	0.1218	1.07 (0.63, 1.82)	0.8030
**Surgery**								
No/unknown	1		1		1		1	
Yes	0.40 (0.32, 0.49)	<0.0001	0.63 (0.48, 0.82)	0.0008	0.43 (0.35, 0.52)	<0.0001	0.64 (0.50, 0.83)	0.0007
**Radiation**								
No/unknown	1		1		1		1	
Yes	1.84 (1.48, 2.29)	<0.0001	1.21 (0.96, 1.53)	0.1066	1.79 (1.46, 2.19)	<0.0001	1.25 (1.00, 1.55)	0.0477
**Chemotherapy**								
No/unknown	1		1		1		1	
Yes	1.24 (0.99, 1.55)	0.0559	0.77 (0.60, 0.99)	0.0435	1.15 (0.93, 1.41)	0.1902	0.76 (0.60, 0.96)	0.0206

**FIGURE 3:**
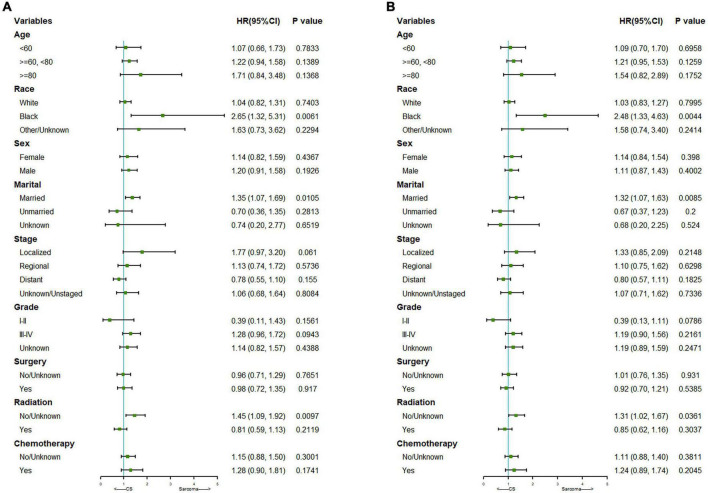
Subgroup analysis for CSS **(A)** and OS **(B)** of the matched PCS and PS patients.

We further did a subgroup survival analysis of the matched PCS and pure PS patients. The results showed that CSS or OS of the two matched cohorts were generally similar in subgroups when stratified by age, gender, staging, grading, surgery, and chemotherapy. However, PCS patients showed significantly worse survival than PS in subgroups of the black race, married status, and no radiation treatment (Figure 3).

### Independent prognostic factors for overall survival of pulmonary carcinosarcoma

We randomly divided 428 PCS patients from SEER into the training set (*n* = 300) and the validation set (*n* = 128) at a ratio of 7:3. Univariate and multivariate Cox regression analyses were performed for OS in the training data set. We found that age, gender, stage, and receiving surgical or radiation treatment were all significantly correlated with OS in patients in univariate analysis. Further multivariable analysis revealed that gender, stage, surgery, radiation, and chemotherapy were independent prognostic indicators for OS (Table 3). These independent prognostic factors were then included in the final nomogram. We found that male patients (HR 1.45; 95% CI: 1.12–1.88; *p* = 0.0046), regional (HR 1.49; 95% CI: 1.00–2.23; *p* = 0.0491), and distant metastasis (HR 3.78; 95% CI: 2.37–6.02; *p* < 0.0001) had the negative impact on the OS. Interestingly, receiving radiation therapy also showed a significantly correlated with poor OS (HR 1.49; 95% CI: 1.10–2.01; *p* = 0.0096). On the other hand, surgery (HR 0.51; 95% CI: 0.37–0.70; *p* < 0.0001) and chemotherapy (HR 0.47; 95% CI: 0.34–0.65; *p* < 0.0001) were associated with favorable OS.

**TABLE 3:** Univariate and multivariate Cox analyses of OS in PCS patients.

Characteristics	Univariable		Multivariable	
		
	HR (95%)	*P*	HR (95%)	*P*
**Age (years)**				
<60	1		1	
60–80	1.33 (1.00, 1.78)	0.0490	1.16 (0.85, 1.59)	0.3338
>80	1.37 (0.84, 2.26)	0.2028	0.98 (0.56, 1.69)	0.9487
**Race**				
White	1		1	
Black	0.92 (0.61, 1.39)	0.7180	0.75 (0.49, 1.16)	0.2027
Other/unknown	0.81 (0.44, 1.50)	0.5146	0.86 (0.46, 1.63)	0.6568
**Gender**				
Female	1		1	
Male	1.32 (1.03, 1.70)	0.0252	1.45 (1.12, 1.88)	0.0046
**Marital status**				
Married	1		1	
Unmarried	0.90 (0.64, 1.27)	0.5726	0.84 (0.58, 1.21)	0.3589
Unknown	1.17 (0.55, 2.48)	0.6803	1.38 (0.62, 3.06)	0.4272
**Stage**				
Localized	1		1	
Regional	1.41 (0.97, 2.05)	0.0677	1.49 (1.00, 2.23)	0.0491
Distant	4.02 (2.74, 5.88)	<0.0001	3.78 (2.37, 6.02)	<0.0001
Unknown	1.30 (0.88, 1.93)	0.1818	1.13 (0.74, 1.72)	0.5672
**Grade recoded**				
I–II	1		1	
III–IV	1.24 (0.54, 2.84)	0.5986	1.14 (0.48, 2.73)	0.7532
Unknown	1.28 (0.56, 2.90)	0.5535	1.14 (0.48, 2.69)	0.7617
**Surgery**				
No/unknown	1		1	
Yes	0.37 (0.28, 0.48)	<0.0001	0.51 (0.37, 0.70)	<0.0001
**Radiation**				
No/unknown	1		1	
Yes	1.92 (1.48, 2.50)	<0.0001	1.49 (1.10, 2.01)	0.0096
**Chemotherapy**				
No/unknown	1		1	
Yes	0.97 (0.75, 1.25)	0.8313	0.47 (0.34, 0.65)	<0.0001

### Establishment and assessment of the nomogram

**FIGURE 4:**
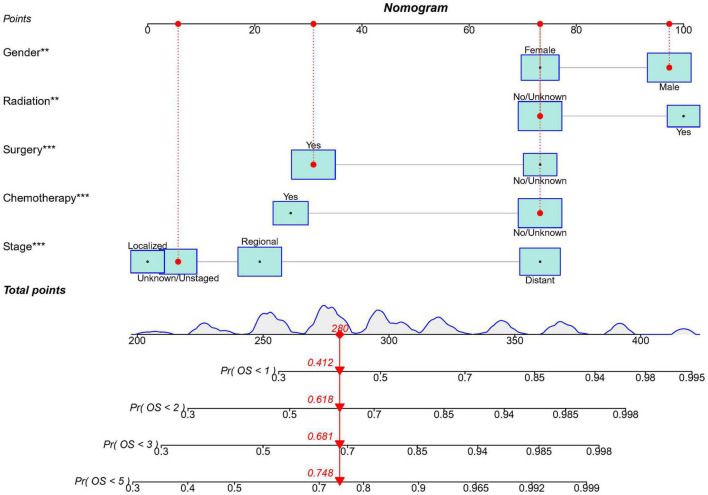
The nomogram model to predict the 1, 2, 3, and 5-years survival probability in PCS patients. ** indicates *p* < 0.01; *** indicates *p* < 0.001.

**FIGURE 5:**
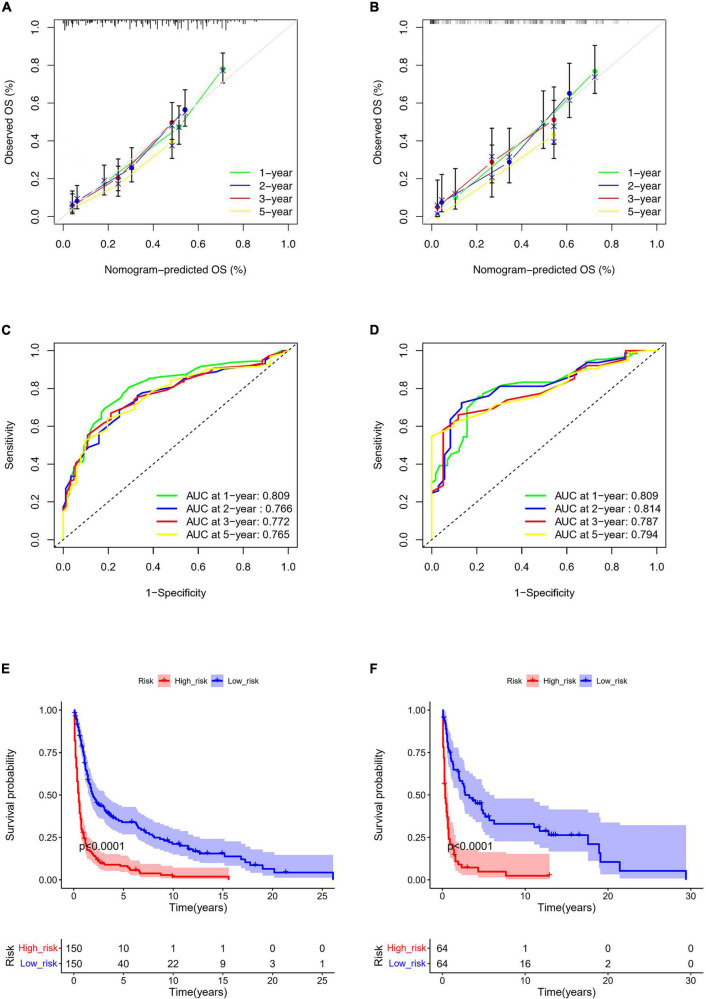
Nomogram calibration curves for predicting OS in the training **(A)** and the validation set **(B)**. ROC curves of the nomogram in the training **(C)** and the validation set **(D)**. Kaplan-Meier curve of the low- and high-risk groups in PCS patients according to the risk scores in the training **(E)** and the validation set **(F)**.

Based on the predictive factors in the multivariable analysis, a nomogram was constructed to predict PCS patients’ 1, 2, 3, and 5-years survival probability (Figure 4). The C-index of the nomogram in predicting OS in the validation cohort was 0.752, which was similar to that observed in the training cohort of 0.720, indicating good discrimination. Then the excellent calibration of the nomogram was observed both in the training and validation sets, with good correlations between predicted and actual observed survival rates (Figures 5A,B). ROC analysis showed the model had good predictive power, with AUCs of 0.809, 0.766, 0.772, and 0.765 at 1, 2, 3, and 5 years in the training group and 0.809, 0.814, 0.787, and 0.794 at different time points in the validation set (Figures 5C,D).

### Risk stratification system

According to the nomogram, we calculated the risk scores of PCS patients in the training and the validation set and divided the two cohorts into low-risk and high-risk groups, respectively. The low-risk group had significantly better survival than the high-risk group both in training (*p* < 0.0001) and the validation set (*p* < 0.0001) (Figures 5E,F). In addition, we used the model to retrospectively analyze the clinical data of 25 PCS patients in the West China Hospital for external validation. Due to the rarity, data of PCS from our hospital can only verify ROC and risk stratification analysis, however, the results still indicate that our nomogram has good performance (Supplementary Figure 1).

## Discussion

Primary PCS, with a mix of carcinomatous and sarcomatous components, represents a rare malignant disease of the lung. Few studies have been reported in the literature, and the estimates about PCS patients’ clinical characteristics and prognosis were imprecise and controversial due to the small and unbalanced statistical sample sizes. PS containing only sarcomatous tissue is a rare lung cancer as well (22). In the present research, we collected the clinical characteristics and survival data of PCS and PS patients from the population-based SEER database and then performed PSM on them to obtain more accurate knowledge about these rare types of lung diseases.

A total of 428 PCS and 249 pure PS patients from SEER were included in our study. We found that 65.8% of PCS were aged 60–80 at diagnosis, roughly consistent with the average age of diagnosis of around 60 reported in previous studies (3, 23). But just 58.6% of PCS patients were men, which is substantially lower than that reported in the literature (13, 16, 24). It is also more male PCS patients than female in our hospital. The SEER database is unavailable for personal smoking history. Thus it is a pity that we can’t confirm the correlation between smoking and PCS patients that is reported in previous literature. In terms of treatment, complete surgical removal of the tumor with negative tumor margins is considered the most desired treatment approach for PCS or PS patients (11, 14, 15, 22). Our results proved that PCS patients receiving surgery (HR 0.51; 95% CI: 0.37–0.70; *p* < 0.0001) or chemotherapy (HR 0.47; 95% CI: 0.34–0.65; *p* < 0.0001) had a better survival. However, receiving radiation therapy showed a significantly correlated with poor OS (HR 1.49; 95% CI: 1.10–2.01; *p* = 0.0096), which means PCS patients are usually resistant to radiation. Compared to patients with pure PS, PCS had a similar proportion of patients who underwent radiation or chemotherapy but more of patients who received surgery (64 vs. 39.3%). The reason for this may be there were more PS patients had unrespectable distant metastasis at diagnosis than PCS (40.5 vs. 26.1%). In recent years, some new treatment approaches, such as targeted or immune checkpoint blockade therapies, have been tested and shown some therapeutic efficacy in PCS patients. Tanimoto et al. reported a case of a patient with metastatic PCS who delivered an excellent response to pazopanib, a multiple kinase inhibitor (25). In addition, Zhang et al. described a case of PCS, with the positive expression of PD-L1, obtained a significant benefit from Nivolumab treatment (26). Of course, these findings need to be further confirmed in extensive sample clinical trials.

The comparison of prognosis between the PCS and PS patients has seldom been reported. One study with 15 PCS and 48 PS patients suggested that the prognosis of PCS patients post-surgery treatment is similar to pure PS patients (13). Another study examining 55 patients with pulmonary sarcomatoid carcinoma (including a small amount of PCS) and 45 patients with PS indicated PS had better survival in resected patients but worse survival in unresected patients (27). Our study is the most extensive PSM retrospective study (1:1 matched; *n* = 232) to date on the prognosis between PCS and PS patients. We found that PCS patients had slightly better survival in the unmatched cohorts whereas non-significantly better survival after PSM. Therefore, the prognosis of the patients with PCS seems to depend on the sarcomatous component of the tumor.

In addition, we constructed a nomogram for PCS based on a population-based database according to all independent prognostic factors, including gender, stage, surgery, radiation, and chemotherapy. It demonstrated good discrimination, calibration, and predictive power in the training and validation cohorts and well-divided PCS patients into low- and high-risk groups. Besides, our model also has good performance in PCS patients from our hospital as external validation. Therefore, we believe this nomogram can provide a more accurate prognosis prediction and help guide the appropriate treatment decisions in clinical practice.

The main limitation of our study is as follows: Firstly, as a retrospective study, there are some inevitable risks of selection bias and confounding factors. Though we have used the PSM method to balance baseline covariates between PCS and PS in our research, it might still influence the accuracy of our results. Secondly, the SEER database is unavailable for more detailed information such as the specific type of carcinomatous or sarcomatous component, specific chemotherapy agents, and genetic mutations. These issues, which can’t be included in Cox regression analyses for PCS patients’ OS, may also affect the prognosis and response to treatment. Thirdly, we analyzed the patients from 1975 to 2018. Over a long time, improvements in surgery, chemotherapy, and targeted or immunotherapies have evolved.

## Conclusion

In summary, PCS is a rare malignant lung disease with distinct clinical features. We discovered that PCS patients’ prognosis was similar to those of pure PS patients. Additionally, we created and validated a prognostic nomogram with good discrimination and calibration for predicting the OS in PCS patients.

## Data availability statement

The original contributions presented in this study are included in the article/Supplementary material, further inquiries can be directed to the corresponding author/s.

## Ethics statement

The studies involving human participants were reviewed and approved by the Ethics Committee of West China Second University Hospital, Sichuan University. Written informed consent for participation was not required for this study in accordance with the national legislation and the institutional requirements.

## Author contributions

M-YZ and AZ contributed to the conception and design of the study. M-YZ, L-ST, Z-JQ, Y-TH, and KC organized the database. M-YZ, L-ST, Z-JQ, and Y-TH performed the statistical analysis. M-YZ, L-ST, and Z-JQ wrote the first draft of the manuscript and wrote sections of the manuscript. All authors contributed to manuscript revision, read, and approved the submitted version.

## Footnotes

1
https://seer.cancer.gov/seerstat

## Supplementary material

Supplementary Figure 1 ROC curve **(A)** and Kaplan-Meier curve of the low- and high-risk groups **(B)** in PCS patients from the West China Hospital.
