# Supplementary figures and images for: Clinical features and prognostic factors of pulmonary carcinosarcoma: A nomogram development and validation based on surveillance epidemiology and end results database
Source: Front Med (Lausanne). 2022 Oct 18;9:988830. doi: 10.3389/fmed.2022.988830 (PMC9622765; doi:10.3389/fmed.2022.988830)

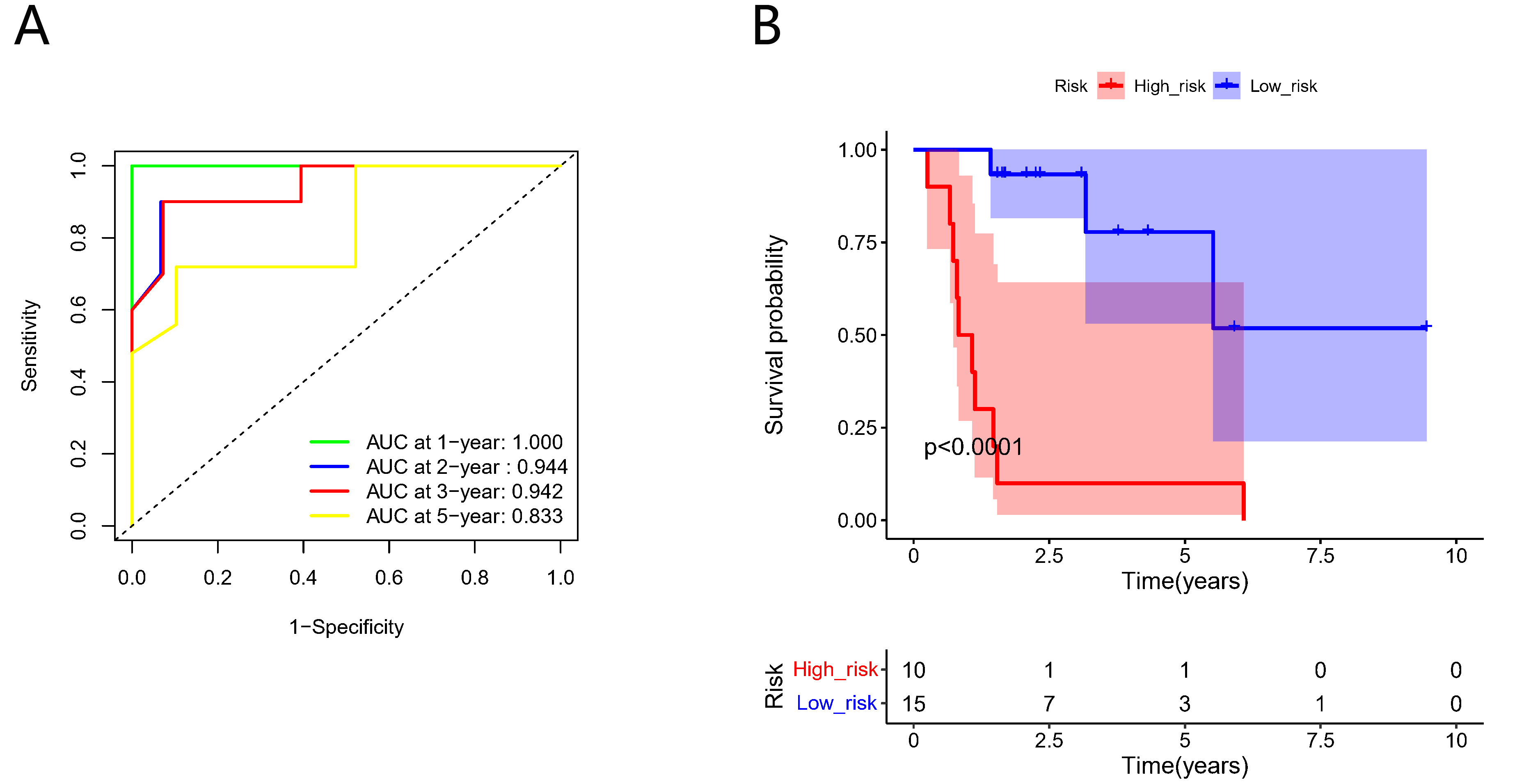

Supplement: Supplementary Figure 1 — ROC curve (A) and Kaplan-Meier curve of the low- and high-risk groups (B) in PCS patients from the West China Hospital. [file Image_1.JPEG]
